# Neuroadaptations in Human Chronic Alcoholics: Dysregulation of the NF-κB System

**DOI:** 10.1371/journal.pone.0000930

**Published:** 2007-09-26

**Authors:** Anna Ökvist, Sofia Johansson, Alexander Kuzmin, Igor Bazov, Roxana Merino-Martinez, Igor Ponomarev, R. Dayne Mayfield, R. Adron Harris, Donna Sheedy, Therese Garrick, Clive Harper, Yasmin L. Hurd, Lars Terenius, Tomas J. Ekström, Georgy Bakalkin, Tatjana Yakovleva

**Affiliations:** 1 The Section of Alcohol and Drug Dependence Research, Department of Clinical Neuroscience, Karolinska Institute, Stockholm, Sweden; 2 Department of Molecular Medicine, Karolinska Institute, Stockholm, Sweden; 3 Waggoner Center for Alcohol and Addiction Research, University of Texas at Austin, Austin, Texas, United States of America; 4 Discipline of Pathology, University of Sydney, Sydney, New South Wales, Australia; 5 Department of Psychiatry, Mount Sinai School of Medicine, New York, New York, United States of America; 6 Department of Pharmacology and Biological Chemistry, Mount Sinai School of Medicine, New York, New York, United States of America; 7 Department of Pharmaceutical Biosciences, Division of Biological Research on Drug Dependence, Uppsala University, Uppsala, Sweden; James Cook University, Australia

## Abstract

**Background:**

Alcohol dependence and associated cognitive impairments apparently result from neuroadaptations to chronic alcohol consumption involving changes in expression of multiple genes. Here we investigated whether transcription factors of Nuclear Factor-kappaB (NF-κB) family, controlling neuronal plasticity and neurodegeneration, are involved in these adaptations in human chronic alcoholics.

**Methods and Findings:**

Analysis of DNA-binding of NF-κB (p65/p50 heterodimer) and the p50 homodimer as well as NF-κB proteins and mRNAs was performed in postmortem human brain samples from 15 chronic alcoholics and 15 control subjects. The prefrontal cortex involved in alcohol dependence and cognition was analyzed and the motor cortex was studied for comparison. The p50 homodimer was identified as dominant κB binding factor in analyzed tissues. NF-κB and p50 homodimer DNA-binding was downregulated, levels of p65 (*RELA*) mRNA were attenuated, and the stoichiometry of p65/p50 proteins and respective mRNAs was altered in the prefrontal cortex of alcoholics. Comparison of a number of p50 homodimer/NF-κB target DNA sites, κB elements in 479 genes, down- or upregulated in alcoholics demonstrated that genes with κB elements were generally upregulated in alcoholics. No significant differences between alcoholics and controls were observed in the motor cortex.

**Conclusions:**

We suggest that cycles of alcohol intoxication/withdrawal, which may initially activate NF-κB, when repeated over years downregulate *RELA* expression and NF-κB and p50 homodimer DNA-binding. Downregulation of the dominant p50 homodimer, a potent inhibitor of gene transcription apparently resulted in derepression of κB regulated genes. Alterations in expression of p50 homodimer/NF-κB regulated genes may contribute to neuroplastic adaptation underlying alcoholism.

## Introduction

Although mechanisms of alcoholism remain to be elucidated, the molecular hypothesis postulates that that alcohol dependence and toxicity result from neuroadaptations to chronic alcohol consumption based on alterations in gene expression. Molecular and cellular adaptations in the nucleus accumbens, ventral tegmental area, amygdala and prefrontal cortex (PFC) [1], [2] have been implicated in the behavioral changes such as craving and relapse induced by chronic alcohol consumption. Chronic alcohol abuse also causes deficits in perceptual-motor skills, visual-spatial functions, abstraction and problem solving [3], [4]. These impairments may be related to alcohol-induced damage to the PFC and hippocampus [5], [6]. White matter and cell loss in the PFC, loss of hippocampal volume and shrinkage of hippocampal neurons are characteristic of these maladaptations [7]–[11].

Earlier work has found that alcohol abuse is associated with widespread changes in gene expression in the PFC of human brain [12]–[14]. Differentially expressed genes form functional groups implicated in immune response, cell survival, inflammation, signal transduction and energy production. Pronounced differences have been found in genes involved in myelination, protein trafficking, apoptosis and neurogenesis [12]–[14]. Re-programming of gene expression in chronic alcoholics apparently involves transcription factors that are responsive to the primary effects of ethanol, and that regulate multiple pathways leading to neuropathology and neuronal dysfunctions.

Transcription factors of the NF-κB family are inducible proteins that regulate expression of genes involved in inflammation, immune response and cell survival [15]–[17]. These factors are homo- or heterodimers of p65 (Rel A), p50 and other proteins of the NF-κB family. The p65/p50 heterodimer (NF-κB) generally activates gene transcription while the p50 homodimer represses it [18], [19]. In most cell types, NF-κB is sequestered in the cytoplasm in a complex with inhibitor IκB proteins. Nuclear translocation of NF-κB is induced by multiple extracellular stimuli that trigger activation of an IκB kinase (IKK) complex, which phosphorylates the IκBs leading to their ubiquitination and proteasomal degradation. The released NF-κB migrates to the nucleus to act as a transcription factor. The IKK complex contains the two kinases IKKα and IKKβ and the regulatory subunit NEMO/IKKγ, and functions as integrator of signals regulating NF-κB activity. Transactivating capacity is also regulated in the cell nuclei through phosphorylation of p65 and p50 by IKKβ and other kinases [20]–[22]. In the brain, the p65 and p50 NF-κB subunits are abundantly expressed in neurons and glia, and a substantial fraction of NF-κB is located in the cell nuclei and constitutively active [15], [16], [23], [24]. The NF-κB-mediated activation of gene transcription contributes to long-term changes in synaptic signaling, cognitive behavior, developmental cell death and chronic neurodegenerative disorders [15], [16], [25]–[31].

Several observations including NF-κB activation by glutamate, cytokines and oxidative stress, ability to transmit signals from the cytoplasm and synapses to the cell nuclei and regulation of synaptic plasticity and neuron survival [15], [16], [22], [25]–[29], suggest a role of this transcription factor in adaptation to chronic alcohol intake. Induction of oxidative stress by alcohol and alcoholism-associated alterations in the expression of inflammatory, cell survival and myelin genes [12]–[14] regulated by NF-κB [15]–[17], [32], [33] support this hypothesis. In the present study, we aimed to evaluate whether the NF-κB system is involved in neuroadaptation of the human brain to chronic alcohol abuse. The PFC involved in alcohol dependence and cognitive behavior, and impaired in human chronic alcoholics was chosen for analysis. The motor cortex (MC) that is not engaged in these processes to the same extent and is less affected by alcohol with respect to neuronal density [8], [10], was studied for comparison. The NF-κB system was characterized with biochemical and molecular techniques, and the alterations found were correlated with changes in the pattern of gene expression previously identified in the PFC of human chronic alcoholics [14].

## Materials and Methods

### Case Selection

Brain samples (superior frontal cortex, Brodmann area 9 and motor cortex, Brodmann area 4) were obtained from the Tissue Resource Center, University of Sydney, Australia (http://www.braindonors.org). A total of 30 cases, all males, including 15 alcoholic and 15 control subjects were analyzed (Table 1). Alcoholic and control cases were matched for age at death, and postmortem interval (PMI). Alcohol subjects met criteria for Diagnostic and Statistical Manual for Mental Disorders, 4th edition, (American Psychiatric Association) [34] and also National Health and Medical Research Council (NHMRC)/World Health Organization criteria as individuals who consumed greater than 80 g of ethanol per day for the majority of their adult lives. Controls had either abstained from alcohol completely or were social drinkers who consumed less than 20 g of ethanol per day on average. Cases with a history of poly drug abuse (evidence that the individual abused other drugs such as cocaine or heroin) or with medical complications such as liver cirrhosis and the Wernicke–Korsakoff syndrome or alcoholic cases with concomitant diseases were excluded. Samples were taken by qualified pathologists under full ethical clearance from the Sydney South West Area Health Service Human Ethics Committee (X03-0117) and informed written consent from the next of kin, kept at −80°C and shipped to Sweden on dry ice.

**Table 1:** Demographic data of alcoholics and control subjects

Subject No.	Age, years	PMI, hs	Brain pH	Cause of death	Toxicologic findings at time of death	Smoking history
**Controls**						
1	34	20.5	6.73	Acute exacerbation of asthma	NA	Smoker
2	78	6.5	6.2	Dehydration and adenocarcinoma of the lung and rectum with multiple metastases	None	Non smoker
3	63	72	6.9	Coronary-artery atherosclerosis	None	Smoker
4	82	23.5	6.4	Sepsis	None	NA
5	38	13.5	6.26	ACSVD	None	Smoker
6	69	16	6.6	ACSVD	Paracetamol, carbon monoxide	Smoker
7	56	24	6.53	Coronary-artery atheroma	NA	Smoker
8	59	20	6.56	Coronary thrombosis	None	Smoker
9	56	25	6.1	CAD	Codeine, morphine, naproxen	NA
10	56	37	6.76	LV scarring, hypertension and cardiomegaly	None	Smoker
11	82	36	6.24	Myocardial infarction	NA	Non smoker
12	44	50	6.6	CAD	None	Smoker
13	51	20	5.88	Cardiac tamponade	None	NA
14	61	24	6.52	CAD	NA	Smoker
15	53	16	6.84	Dilated cardiomyopathy	Lignocaine, sotalol	Non smoker
**Mean±S.E.M.**	**59±1**	**27±1**	**6.47±0.02**			
**Alcoholics**						
1	34	8.5	6.61	Hanging	Alcohol	Smoker
2	77	20	6.34	Lobular pneumonia	None	Smoker
3	65	32	5.66	Seizures and cardiac arrest	Codeine, moclobemide, paracetamol, phenytoin, quinine	NA
4	79	48	6.34	CAD	Temazepam	Smoker
5	39	24	6.56	Aortic sclerosis	NA	Smoker
6	70	33.5	6.24	Respiratory failure	None	Smoker
7	56	45	6.51	BEV	Alcohol	NA
8	59	24	6.57	Cardiomyopathy	None	Non smoker
9	56	22	6.52	CAD and UGIH	None	Smoker
10	56	15	6.66	CAD and emphysema	Nordiazepam	NA
11	81	36	6.44	Sepsis	None	Smoker
12	44	15	6.48	CAD	Diazepam, noridazepam	Non smoker
13	52	45.5	6.78	Lobar pneumonia	None	Smoker
14	62	49	6.49	CAD	Sertraline	Smoker
15	53	57	6.75	CAL	NA	Smoker
**Mean±S.E.M.**	**59±1**	**32±1**	**6.46±0.02**			

Abbreviations: ACSVD, atherosclerotic cardiovascular disease; BEV, bleeding esophageal varices; CAD, ischaemic heart disease; CAL, chronic airflow limitation; LV, left ventricular; NA, not available; PMI, postmortem interval and UGIH, upper gastrointestinal hemorrhage. Smoker group includes smokers, ex-smokers (subjects ## 3, 12 and 14 in controls, and 11 and 14 in alcoholics) and occasional smoker (subject #1 in controls).

### mRNA quantification by TaqMan® low density array

RNA preparation was performed using RNeasy Lipid Tissue Mini Kit (QIAGEN, Maryland, USA). RNA was quantified by Nanodrop® and Agilent 2100 Bioanalyzer (Agilent, Palo Alto, CA) was used to control RNA quality. Only RNA with clear ribosomal RNA, 18S and 28S, was used for further analysis. cDNA was synthesized with the High Capacity cDNA Archive Kit (Applied Biosystems, Foster City, CA). mRNA levels were quantified by TaqMan® Low Density Arrays (Applied Biosystems, Foster City, CA). In a pre-prepared micro fluidic card containing probes and primers for each gene, cDNA and TaqMan® Universal PCR Master Mix (Applied Biosystems) was added in a final concentration of 65 pg cDNA per sample and gene. Every sample was run in duplicate on the same array for each gene. The PCR amplification was performed at 50°C for 2 min, 94. 5°C for 10 min and 40 cycles of 97°C for 30 seconds followed by 59.7°C for 1 min. To measure the quantity of a given RNA species, the threshold cycles (Ct) were monitored by the Applied Biosystems 7900HT Fast Real-Time PCR System. Each mRNA expression was calculated by relative quantification using a normalization factor (geometric mean of two reference genes selected by geNORM program, http://medgen.ugent.be/genorm/) [35] and qBASE program for internal and external calibration and also for easy care of large RT-PCR datasets (http://medgen.ugent.be/qbase/). According to our analysis of reference genes [36] the beta-actin (ACTB) and ribosomal large P0 (RPLP0) genes for PFC, and the 18S and RPLP0 genes for the MC were chosen for normalization.

### Western Blot Analysis

Powdered tissue samples were boiled in pre-warmed SDS extraction buffer (0.45 M Tris-HCl, pH 8.5, 2.5% glycerol, 4% SDS, 0.5 mM DTT and 5×protease inhibitors for 5 min, sonicated and protein extracts were aliquoted and kept at −80°C until usage. Protein concentration was determined with the *DC* protein assay (Bio-Rad Laboratories, Hercules, CA, USA). Aliquots of tissue extracts (50–120 µg protein) were heated for 5 min at 95°C in the presence of 5 mM β-mercaptoethanol and resolved by SDS-PAGE on 10% Tricine gels as described earlier [37]. Reference samples consisting of pooled cerebellar extracts from control subjects were loaded onto three wells, on each edge and in the middle of each gel and their density values were used to ascertain reproducibility on each blot and interblot comparison. Proteins were transferred onto nitrocellulose membranes (Schleicher and Schuell, Dassel, Germany) at 4°C and stained with Memcode Reversible Protein Stain Kit (Pierce, Rockford, USA). Densitometry values of protein load were used for normalization of Western blot data. After destaining, membranes were incubated with stripping buffer (62.5 mM Tris-HCl, 2% SDS, 100 mM β-mercaptoethanol, pH 6.7) for 20 min at 55°C, blocked for 30 min with 5% non-fat dry milk in 50 mM Tris-HCl, 0.15 M NaCl, 0.05% Tween 20 buffer and probed with rabbit polyclonal antibodies against the human NF-κB p50 or p65 subunits (PC136, 1∶2000; PC137, 1∶2000; Calbiochem, San Diego, CA, USA), or goat polyclonal antibodies against the C-terminus of human IKKβ (sc-7329; 1∶500; Santa Cruz, California, USA). Goat anti-rabbit (Bio Rad) or rabbit anti-goat (Sigma) antibodies all conjugated with horseradish peroxidase (HRP) were used as secondary antibodies. Membranes were developed with the ECL detection system (Amersham, Little Chalfont, UK). Fuji Film Image Gauge software was used for densitometric analysis.

### NF-κB DNA binding activity

#### Preparation of whole tissue extracts

All experimental procedures were carried out at +4°C. Tissue extracts were prepared by a modification [24] of the protocol described elsewhere [38], [39]; powdered tissue preparations (25–35 mg) were homogenized in 4 volumes of Buffer C (20 mM HEPES, pH 7.9, 0.42 M NaCl, 25% glycerol, 1.5 mM MgCl_2_, 0.4 mM EDTA, 0.5 mM DTT, 0.2% NP-40, 25 µM proteasome inhibitor MG132 and 5×protease inhibitors (Complete, Roche, Switzerland)) by 10 strokes with plastic pestles (Sigma-Aldrich, St. Louis, MO, USA) in Eppendorf tubes, incubated for 10 min, homogenized again by 10 strokes and centrifuged at 20,000×g for 15 min at 4°C. The supernatant was kept at −80°C until assayed. Protein concentration was determined using the *DC* protein assay.

#### Oligonucleotides

The following oligonucleotides were SDS-PAGE purified and [P^32^] labeled with Klenow enzyme as described earlier [24]. Sequence of the plus strand of wild type (wt) and mutant (m)-κB fragments of the immunodeficiency virus enhancer [40] is shown; wt- and m-κB-sites are underlined and mutated nucleotides are indicated by bold letters: wt-κB 5′-GGTGATCAGGGACTTTCCGCTGGGGACTTTCCAGGAT-3′, m-κB 5′-GGTGATCA**TTC** ACTTTCCGCT**ATTC** ACTTTCCAGGAT-3′. Minus strands were extended 1 to 3 nucleotides from the 5′-end and recessed by 1-3 nucleotides from the 3′-end.

#### Electrophoretic mobility shift assay (EMSA)

The EMSA was performed essentially as described previously [24]. Protein extracts (10 µg) were added to the binding mixture (20 µl of 10 mM Tris-HCl, pH 7.5, 20 mM KCl, 1 mM EDTA, 7.5% glycerol, 1.5 mM DTT, 20 µg BSA and 40–60,000 cpm [^32^P] –labeled oligonucleotide) in duplicates and incubated for 20–30 minutes at room temperature. The reaction mixture was brought to 0.6% or 0.03% deoxycholate (DOC) after 30 min followed by the addition of 1.2% NP-40 and were loaded onto a 5% polyacrylamide gel in 0.5×TGE (25 mM Tris-HCl, 0.19 M glycine, 1 mM EDTA, pH 8.5) buffer. Reference samples consisting of pooled cerebellar extracts from control subjects were loaded onto two wells on each edge of the gel to ascertain reproducibility of the gel, and also for inter-gel comparison. In competition experiments, 10 ng of unlabeled wt-κB or m-κB oligonucleotide was added to the incubation mixture prior to the initiation of the binding reaction. After electrophoresis the gels were fixed in 40% methanol containing 3% acetic acid for 10 min, dried and exposed to intensifying screen. Detection was performed on phosphorimager BAS 1500 (Fuji Film, Kanagawa, Japan) and densities were analyzed using Fuji Film Image Gauge software.

### Computational Analysis

NF-κB- and p50 homodimer DNA-binding sites in genes differentially expressed in the PFC of human alcoholics compared to control subjects [14] were analyzed with the Prometheus system [41]. Transcription Factor (TF) Binding Sites (TFBS) were identified in phylogenetically conserved (human versus rat) regions of the 10 kb-promoter region. The weight matrices for TFBS identification were taken from JASPAR [42]. Prometheus uses the GeneLynx Database [43] and the GeneLynx application programming interface to obtain the genomic coordinates and sequences for human and rat species with an upstream promoter length of 10 kb. LAGAN aligner algorithm was run using a max gap length of 5 bp, a conservation rate of 45 bp, conservation super rate of 65 bp and a score cutoff of 25 bp [44], [45]. The TFBS finding algorithms were run using a window size of 50 bp, a conservation cutoff of 70% and a TF score threshold of 80% for human-rat comparison. The system uses the Grid computing paradigm [46] for the execution of computationally intensive steps involved in the TFBS finding process: genomic sequence alignment and TFBS searching. The Grid computational resources are provided by NorduGrid Virtual Organization (http://www.nordugrid.org/). Significance of differences in a number of TFBS between gene sets was evaluated with the Chi-Square test. 479 out of 531 differentially expressed genes identified by Liu et al. [14] were analyzed (Table supplemental (S) 1); 52 out of the 531 genes were not included because of missing annotations/sequences. Brain autopsy material used in the present and Liu et al. [14] studies was partially obtained from the same source (Tissue Resource Center, University of Sydney); only two of all subjects were analyzed in both studies. Sets of downregulated and upregulated genes were compared with each other, and with a control set of 1164 genes (Table S1). Control set did not include genes differentially expressed in the PFC of alcoholics.

### Statistical Analysis

Results are expressed as mean and standard deviation (normally distributed variables) or median and range (non-normal distribution). Normality of data distribution was analyzed using Shapiro-Wilk's W-test. In data sets that were not normally distributed non-parametric analysis (Mann-Whitney and Spearman correlations) was used. A general stepwise linear regression model was used to evaluate group differences and identify covariates (age, brain pH and postmortem interval). Student's *t*-test was used to assess differences between groups when no covariates were found. Variables with a significant association with group were included in the final statistical model as covariates. Pearsons correlation analysis was performed to determine the association between different variables. Slopes in the correlation equation were compared with a multiple regression model. Differences in the number of up-, down-regulated and control genes containing TFBS were evaluated with Chi-Square test. Significance was set at P<0.05 and trends considered for P<0.10. Statistical analysis was carried out using the Statistica 6.0 package (StatSoft Scandinavia, Uppsala, Sweden).

## Results

The demographic characteristics of control and alcoholic subjects are given in Table 1. We found no significant differences in age (t_28_ = 0.01, P = 0.99), PMI (t_28_ = 0.85, P = 0.40) and proportions of smokers and nonsmokers (Fisher test, P = 0.5) between controls and alcoholics. All subjects were Caucasian male. Brain pH was determined to assess the agonal state. The mean brain pH of controls and alcoholics were 6.46±0.29 and 6.47±0.27, respectively, which was not significantly different (t_28_ = 0.11, P = 0.91).

### mRNA expression levels of NF-κB related genes

*RELA, NFKB1, NFKB2, RELB, IKKβ* and *IκBα* mRNAs that give rise to p65, p50, p52, Rel B, IKKβ and IκBα proteins were quantified in the PFC (Brodmann area 9) of human chronic alcoholics and control subjects (n = 15/group), and in the MC (Brodmann area 4) of the same subjects using TaqMan® Low Density Arrays (Table 2; n = 15/group). In the PFC, *RELA* mRNA was significantly decreased by 1.41 fold in alcoholics compared to the control group (stepwise linear regression F_2,27_ = 10.03, P = 0.003, covariate pH). No group×pH interaction was observed. The levels of the other five mRNAs did not differ significantly between alcoholics and controls. In the MC, a trend for an increase in the *RELB* mRNA levels in alcoholics (Student's *t*-test, T_1,28_ = 1,89, P = 0.069) was observed whereas the levels of other five mRNAs showed no significant differences between the groups (Table S2).

**Table 2:** Levels of the NF-κB and p50 DNA-binding, proteins and mRNAs in the PFC of alcoholics and control subjects

	Control group	Alcohol group	P-value
	Mean±SD	Mean±SD	
**mRNA levels**			
*RELA*	2.59±0.97	1.84±0.74	0.003 ^b^
*NFKB1*	2.18±0.87	1.79±0.58	0.15 ^a^
*IKKβ*	2.09±0.81	1.70±0.53	0.14^ a^
*IκBα*	2.48±1.24	1.87±1.04	0.11 ^c^
*NFKB2*	2.64±1.31	2.41±1.27	0.95 ^c^
*RELB*	2.19±1.03	2.48±1.69	0.81 ^a^
**Protein levels**			
p65	0.81±0.36	0.68±0.40	0.206 ^c^
p50	0.81±0.33	0.95±0.37	0.269 ^a^
IKKβ	0.68±0.17	0.68±0.26	0.947 ^a^
**DNA binding activity**			
NF-κB (Act)	67.3±27.2	46.4±13.9	0.037 ^a^
NF-κB (Tot)	63.3±18.7	42.3±22.5	0.010 ^c^
(p50)_2_	72.1±22.9	53.5±12.5	0.029 ^a^

mRNA levels are presented as normalized relative expression levels. Protein levels are presented as relative values calculated from the optical density of p65, p50 or IKKβ bands as a percent from internal control samples and normalized to Memcode staining. Protein and mRNA levels were analyzed in 15 control subjects and 15 alcoholics. NF-κB (Act), constitutively active NF-κB; NF-κB (Tot), total NF- κB activated by 0.6% DOC. Values for DNA binding activity are presented as percent of those obtained with the internal control samples loaded on each gel (control group: n = 11; alcoholic group: n = 13). Significance of differences between groups was evaluated by ^a^ Student's *t*-test, ^b^ covariance by multiple regression analysis (*RELA,* covariate pH), or ^c^ Mann Whitney U-test dependent on the normality of data distribution.

### p65, p50 and IKKβ protein levels

**Figure 1:**
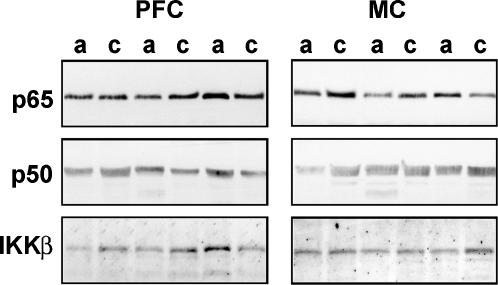
WB images of p65, p50 and IKKβ proteins in the PFC. Representative blots of three control subjects (c) and three alcoholics (a).

Levels of p65 and p50 NF-κB subunits and IKKβ protein were examined by western blotting. Each protein analyzed was identified as a single band with the predicted molecular size–65 (p65), 50 (p50) and 87 (IKKβ) kDa (Figure 1). The optical density values obtained for the proteins were within the linear range of detection as was evident from experiment with serial dilutions of reference samples; densitometry assessment of bands showed linear dependence with correlation coefficient equal to 0.998 between protein immunoreactivity measured as optical density and protein load measured as Memcode optical density (see also [37]). For inter-gel comparison, the optical density of proteins in each sample was expressed as a percentage of reference samples on a given gel, and these values were used for statistical analysis. Similar to the levels of *RELA* mRNA, the p65 levels in the PFC of alcoholics were decreased by 1.46-fold (Table 2) but the differences did not reach statistical significance. The levels of p65 in the MC, and those of p50 and IKKβ proteins in neither the PFC (Table 2; n = 15) nor MC (Table S2; n = 15) differed significantly between control subjects and alcoholics. We were unable to analyze other NF-κB related proteins including cREL, IκBα, IKKα and IKKγ in postmortem human brain samples due to the absence of specific and sensitive antibodies suitable for such study or low levels of expression.

### Stoichiometry of NF-κB proteins and mRNAs

**Figure 2:**
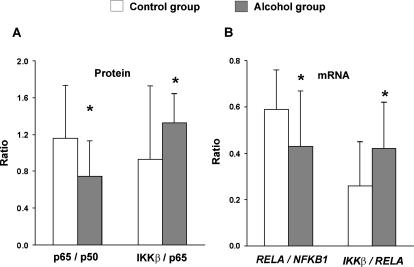
Stoichiometry of p65/p50 and IKKβ/p65 proteins (A), and *RELA*/*NFKB1* and *IKKβ*/*RELA* mRNAs (B). Results are presented for the PFC of alcoholics (n = 15) and control subjects (n = 15) as mean±SD. Significance of differences between groups was evaluated by Mann Whitney U-test (A, p65/p50), covariance by multiple regression analysis (A, IKKβ/p65, covariate age; age×group interaction), or Student's *t*-test (B). * P<0.05.

To test whether stoichiometry between components of the NF-κB system was altered in alcoholics we analyzed the ratio of levels of p65/p50, IKKβ/p65 and IKKβ/p50 proteins and respective mRNAs. The p65/p50 protein ratio was significantly decreased (1.23-fold; Mann Whitney U-test, Z_1,28_ = 2.38, P = 0.017) while the IKKβ/p65 protein ratio increased (1.44-fold; stepwise linear regression, F_2,27_ = 5.78, P = 0.023, covariate age) in the PFC of alcoholics (Figure 2A; n = 15). There was a significant age×group interaction with a negative correlation to age in both groups.

The *RELA/NFKB1* and *IKKβ*/*RELA* mRNA ratio was significantly decreased (1.37-fold) or increased (1.62-fold), respectively, in alcoholics compared to control subjects (Figure 2B; n = 15/group; Student's *t*-test, T_1,28_ = 2,18, P = 0.038 and T_1,28_ = 2,21, P = 0.035). These differences were not due to lower levels of *RELA* mRNA (1.41-fold) in alcoholics because the levels of *NFKB1* and *IKKβ* mRNAs were also decreased by 1.22 and 1.23-fold, respectively, in this group. No significant differences in these protein and mRNA ratios were observed in the MC. Changes in mRNA stoichiometry may result in the disproportion in p65 and p50 protein content affecting formation of NF-κB complexes, and altered IKKβ-mediated p65 phosphorylation.

### NF-κB DNA binding activity

**Figure 3:**
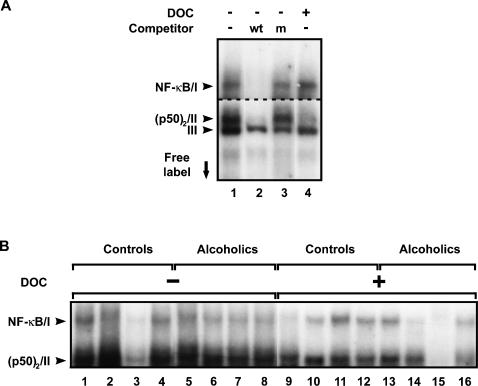
κB–binding factors in the PFC identified by EMSA. (A) The constitutively active κB-DNA binding activity was determined by incubation of tissue extracts with labeled κB-oligonucleotide followed by treatment with 0.03% DOC solution (DOC-) that did not affect DNA-protein complex formation and was used as a control. Latent factors were activated for binding to DNA by treatment with 0.6% DOC (DOC+) that dissociated IκB inhibitory protein from complexes with NF-κB, thus allowing to measure the total DNA-binding. Total NF-κB activity consisted of the constitutively active and DOC-activated DNA binding activities. The specificity of DNA-protein complexes was assessed by competition with wild type-κB (wt) or mutant-κB (m) oligonucleotides. Upper sequence-specific complexes I and II consisted of NF-κB and p50 homodimer, respectively, as demonstrated previously [24], [47]. The lower complex III showed no DNA-binding sequence specificity and was probably formed by Ku protein [48], [49]. For the upper image, film was exposured with a gel for longer time than for the lower one. (B) Representative picture of the constitutively active (DOC-) and total (DOC+) κB–binding activities in the PFC of control subjects and alcoholics. Lanes 3 (subject 13C) and 15 (subject 3A), only weak or no complexes were formed (see Table 1 for the description of subjects).

NF-κB DNA-binding activity was compared in alcoholics and controls using EMSA. The assay identified three main complexes in both PFC and MC (Figure 3A). Two upper complexes (I, II) were specific since their formation was inhibited in the presence of unlabeled wt-κB oligonucleotide (Figure 3A, lane 2) but not m-κB oligonucleotide (Figure 3A, lane 3). Formation of the third, lower complex (III) was not blocked by wt- or m-κB oligonucleotide demonstrating low specificity and affinity of this factor for binding to DNA. We previously identified protein factors in the complexes I and II as NF-κB (p50/p65 heterodimer) and p50 homodimer, respectively, using the supershift/depletion assay with anti-p65- and p50-antibodies and also UV-crosslinking in extracts of human neuronal cell lines and rat brain [24], [47]. We also identified complex III as the Ku protein that binds to double-stranded DNA ends, does not recognize specific DNA sequences, and is present at high levels in the human brain [48], [49]. The levels of the constitutively active and total (active plus latent) NF-κB DNA-binding were measured in the presence of 0.03% and 0.6% DOC, respectively; at high concentration DOC dissociates complexes of NF-κB with the IκB inhibitory protein [24], [38], [47]. The NF-κB/complex I was induced by pre-incubation of extracts from the PFC of control subjects or alcoholics with 0.6% DOC only by 12% and 14%, respectively; thus NF-κB was virtually present in the activated form in this structure (Figure 3A, lane 4). 0.6% DOC slightly inhibited p50 homodimer binding to DNA. The p50 homodimer was a dominant κB binding factor in the human cortex; the levels of its DNA-binding activity exceeded those of NF-κB by 2.4–2.7 fold in the PFC and MC of controls and alcoholics (Student's *t*-test for dependent samples: PFC/controls, T_1,10_ = 8.56, P<0.0001, n = 11; PFC/alcoholics, T_1,12_ = 12.77, P<0.0001, n = 13 (see Figure 3B, lines 1, 2, 4–8); MC/controls, T_1,10_ = 10.83, P<0.0001, n = 11; MC/alcoholics, T_1,12_ = 8.48, P<0.0001, n = 13).

DNA-binding complexes were identified in the PFC and MC in 24 out of 30 subjects analyzed. In contrast, the NF-κB, p50 homodimer and Ku DNA-binding activity was much lover or virtually undetectable in extracts from other 4 control and 2 alcoholic subjects (Figure 3B, lanes 3 and 15). Stepwise linear regression identified pH (p<0.05) as a covariate for the DNA binding activities in the PFC and MC when 30 subjects were analyzed. The six subjects, which extracts failed to produce complexes, had the lowest brain tissue pH ranging from 5.66 to 6.24; the average brain pH for the remaining subjects was 6.61±0.19 and 6.54±0.14 for the control and alcoholic groups, respectively. Thus, DNA binding proteins including NF-κB, p50 homodimer and Ku were probably inactivated due to the low pH level of the sample. When these six subjects were excluded from the analysis, the remaining samples showed no correlation between pH and DNA binding activity.

The p50 homodimer DNA binding activity (Table 2; Student's *t*-test with unequal variances; T_1,14.9_ = 2.41, P = 0.029), and the constitutively active and total NF-κB DNA-binding activity (Student's *t*-test with unequal variances; active, T_1,14.3_ = 2.31, P = 0.037; total, Mann-Whitney U test: Z_1,22_ = 2.58, P = 0.01, respectively), were lower in the PFC of alcoholics compared to controls. No significant differences between alcoholics and controls in the DNA binding activity of p50 homodimer and constitutively active NF-κB were found in the MC although a trend (Student's *t*-test; T_1;22_ = 2.02, P = 0.055) for decrease of the total NF-κB DNA binding activity was observed in alcoholics (Table S2).

### Correlations

**Figure 4:**
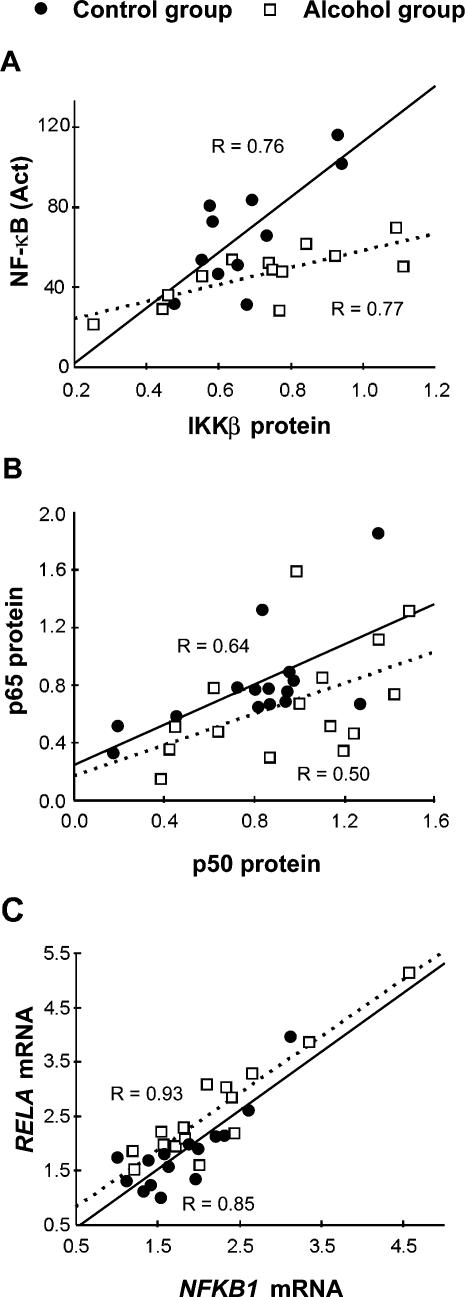
Correlations between DNA-binding activity, protein and mRNA levels. (A) Constitutively active NF-κB DNA binding correlates with IKKβ protein levels in the PFC of alcoholics (n = 13; P = 0.002) and control subjects (n = 11; P = 0.007). No significant difference was found between slopes in the alcohol and control group. (B) p65 and p50 protein levels (15 control subjects: P = 0.01; 15 alcoholics: P = 0.06) and (C) *RELA* and *NFKB1* mRNA levels (15 control subjects: P<0.0001; 15 alcoholics: P<0.0002) correlate in the PFC.

P and r-values of numerically significant correlations are shown in the Table S3. DNA-binding activity of constitutively active NF-κB positively correlated with IKKβ protein content in the PFC of both alcoholics and control subjects (Figure 4A). The p65 and p50 protein levels positively correlated in the PFC of the control group while the correlation was weaker in alcoholics (Figure 4B). Correlations between the six mRNAs analyzed were evaluated in each cortex and for alcoholics and control subjects, respectively (Table S3). Four correlations in the PFC of both alcoholics and control subjects had r-values that exceeded 0.8 and P<0.0001; *RELA* vs. *NFKB1* (Figure 4C), *RELA* vs. *IKKβ, NFKB1* vs. *IKKβ* and *RELA* vs. *IκBα* (Table S3). The interrelationship of the six NF-κB mRNA levels and levels of transcripts of the tumor necrosis factor receptor 1A, p53, myelin basic protein and DNA fragmentation factor α genes in the PFC were evaluated for comparison; no or poor correlations were found with most of them having r-values ranging from 0.1 to 0.6.

### NF-κB- and p50 homodimer-binding sites in genes differentially expressed in alcoholics

**Figure 5:**
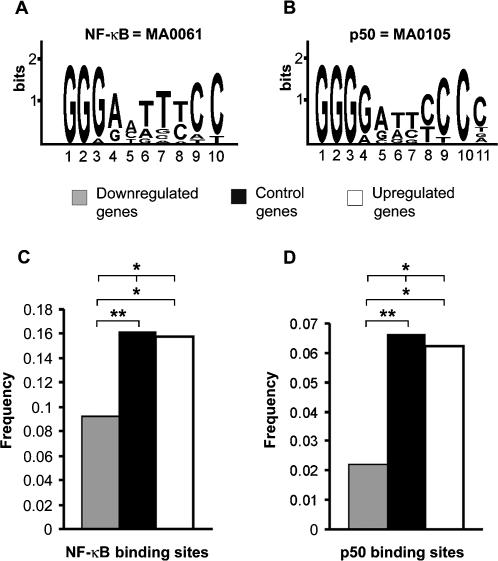
κB elements in genes differentially expressed in alcoholics. Frequencies of occurrence of the “NF-κB”- and “p50 homodimer”-subtypes of κB binding sites in 270 and 209 genes downregulated and upregulated in alcoholics, respectively, and 1164 control genes (Table S1). (A,B) Matrices from the JASPAR database (http://mordor.cgb.ki.se/cgi-bin/jaspar2005/jaspar_db.pl) used to identify “NF-κB” (A) and “p50 homodimer” (B) subtypes of κB binding sites. Both NF-κB and p50 homodimer bind to the “NF-κB” subtype of κB elements with high affinity and specificity whereas p50 homodimer has higher affinity for more symmetric “p50 homodimer”-subtype of κB binding sites [51], [52]. The matrix logos provide visual representation profiles of nucleotide conservation in κB elements. The maximal conservation amounts are 2 bits for each position. Higher conservation indicates increased biological importance for a base. (C, D). Frequencies of occurrence of the “NF-κB”- (C) and “p50 homodimer”- (D) subtypes of κB binding sites in 270 and 209 genes downregulated and upregulated in alcoholics, respectively, and in 1164 control genes (Table S1). Significance of differences between upregulated, downregulated and control genes in the frequency of occurrence of genes with κB elements were evaluated with Chi-square test (df = 2). Pairwise comparison of the up- with downregulated genes, the upregulated with control genes, and the downregulated with control genes was performed with Chi Square test (df = 1). ** P<0.01, * P<0.05.

Downregulation of DNA-binding of the p50 homodimer and NF-κB in alcoholics may result in changes in expression of genes that are regulated through κB elements, binding sites for these transcription factors. To test this hypothesis we used a computational approach to compare the number of genes containing κB elements in two sets of genes that were previously identified by one of the co-author groups [14] as downregulated (n = 270) or upregulated (n = 209) in the PFC of alcoholics (Table S1). A group of 1164 genes that were randomly selected from the set, which did not include genes differentially expressed in alcoholics, was studied for comparison (control genes; Table S1). Previous analyses human chromosome 22 with chromatin immunoprecipitation and genomic microarrays [50] demonstrated that κB elements are enriched at the 5′-end of genes. Therefore, we focus on the identification of κB elements in 10 kb-promoter regions. p50 homodimer and NF-κB bind to virtually the same κB elements. Weight matrix for NF-κB in the JASPAR database [42] consists of sequences of the “NF-κB”-subtype that are also targets for the p50 homodimer (Figure 5A). p50 protein binds to κB sites as homodimer and therefore has slightly higher affinity for symmetric κB elements [51], [52]; this is reflected in the composition of the second κB matrix of the “p50 homodimer”-subtype present in the JASPAR database (Figure 5B). We used both matrices in the analysis (Figure 5). To increase prediction specificity we used a phylogenetic footprinting approach based on the observation that functional Transcription Factor Binding Sites (TFBS) are more often located in evolutionary conserved regions [53], [54]. Analysis of the 479 differentially expressed genes with phylogenetic footprinting of human-rat gene sequences demonstrated that 58 genes contain one or more putative κB binding sites for both p50 homodimer and NF-κB (the “NF-κB”-subtype), and 19 genes contain at least one putative “p50 homodimer”-subtype κB binding site (Table S4). Among 1164 control genes, 188 genes contain putative “NF-κB”-subtype whereas 77 genes putative “p50 homodimer”-subtype of κB binding sites.

Next, we evaluated differences in the proportion of genes that contain the “NF-κB”- or “p50 homodimer”-subtypes of κB elements in 10 kb promoter region between upregulated, downregulated and control genes (Figure 5C, D; Table S4). There was a significant difference in the frequency of occurrence of genes with each “NF-κB”- or “p50 homodimer”- subtype of κB elements between up- and down regulated genes and control genes (Chi-square test, df = 2: *χ^2^* = 8.30, P<0.05; *χ^2^* = 7.75, P<0.05). The frequency of each subtype of binding sites in the downregulated genes was lower compared to the upregulated genes (Chi-square test, df = 1: *χ^2^* = 4.72, P<0.05; *χ^2^* = 4.94, P<0.05) and control genes (Chi-square test, df = 1: *χ^2^* = 8.23, P<0.01; *χ^2^* = 7.75, P<0.01) (Figure 5 C, D). Thus, genes with κB elements were generally upregulated in the PFC in alcoholics; these genes may be derepressed due to decreased activity of the p50 homodimer, an inhibitor of transcription and a dominant κB binding factor.

## Discussion

**Figure 6:**
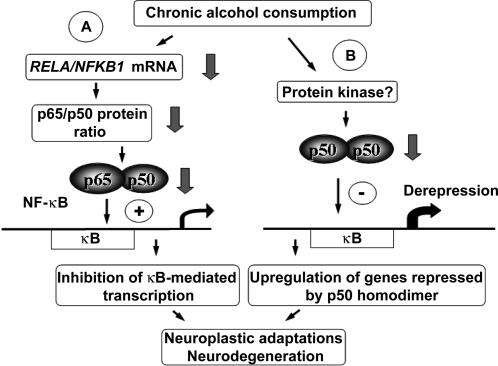
Model for the molecular adaptations in the NF-κB system in the PFC in alcoholics. (A) Chronic alcohol intake inhibits *RELA* gene transcription and induces alterations in stoichiometry of *RELA/NFKB1* mRNAs and p65/p50 proteins, resulting in downregulation of NF-κB DNA-binding. In cells with dominant NF-κB this adaptation results in inhibition of NF-κB-dependent transcription. (B) Alcohol acts upstream of an unidentified protein kinase that modulates DNA-binding of the p50 homodimer. This results in hypophosphorylation of p50 and downregulation of p50 homodimer DNA-binding. In control individuals the p50 homodimer inhibits gene transcription acting through κB binding sites in cells in which it prevails over NF-κB. In alcoholics, downregulation of the p50 homodimer eliminates transcriptional repression leading to upregulation of κB-mediated transcription. Dysregulation of the NF-κB/p50 homodimer-dependent gene transcription contributes to cellular neuroplastic adaptations and neurodegeneration.

The main findings of the present study are that DNA-binding of NF-κB and p50 homodimer as well as *RELA* gene expression are downregulated in the PFC of chronic alcoholics. The downregulation of DNA-binding might result from the decrease in *RELA* gene expression, and from the alterations in stoichiometry in p65, p50 and IKKβ proteins (Figure 6). p65 and p50 proteins constitute NF-κB and p50 homodimer while IKKβ integrates signals regulating the NF-κB system through phosphorylation of IκBα and p65. No significant differences between control and alcoholic groups in the NF-κB system were found in the MC suggesting that dysregulation of NF-κB-mediated adaptive mechanisms is specific for the PFC.

Several factors may affect the quality of RNA and proteins, and DNA binding activities of transcription factors in postmortem human tissues. In the present study, the RNA quality was controlled and no samples with degraded RNA were used in the analysis. No degraded proteins seen as smearing or additional bands on western blots were evident in any sample. Using our protocol for fast extraction of nuclear proteins from frozen tissues, we observed similar levels of the NF-κB/p50 homodimer DNA binding activities in extracts of frozen human samples and of rat brain tissues prepared immediately after decapitation, thus demonstrating low if any inactivation of these factors in human samples. Six subjects demonstrated no NF-κB, p50 homodimer and Ku protein DNA binding activity were characterized by low brain pH values and were omitted from the analysis. Levels of RNA, proteins and NF-κB/p50 homodimer DNA-binding did not significantly correlate with postmortem interval and age at death of subjects analyzed suggesting that influences of these covariates on studied molecular parameters are negligible.

NF-κB is classically activated through dissociation from IκB in the cytoplasm [15]–[17], [20]–[22], [51]. DNA-binding and transactivation functions are also regulated through posttranslational modifications of NF-κB proteins in the cell nucleus. Phosphorylation at several sites by the IKK complex increases the DNA binding and transcriptional activities of p65 [20], [51], [55]. p50 phosphorylation by protein kinase A and other kinases is critical for binding of the p50 homodimer to DNA, which results in transcriptional repression of NF-κB dependent genes [18], [19]. In the apparent absence of changes in the p50 content, the downregulation of p50 homodimer DNA-binding in chronic alcoholics may occur due to the deficient phosphorylation of this protein (Figure 6).

The p50 homodimer is capable of suppressing the transactivation activities of NF-κB by occupying the same κB sites and recruiting corepressor complexes containing histone deacetylases (HDAC) [56]. We found that the p50 homodimer is the dominant κB binding factor in the human PFC. Bioinformatic analysis demonstrated that the proportion of genes with putative κB elements was significantly higher in the set of genes upregulated in the PFC of alcoholics compared to the set of downregulated genes. The most plausible explanation for these observations is that in alcoholics the p50 homodimer-induced repression of gene transcriptions weakens due to the p50 homodimer downregulation thus allowing the recruitment of NF-κB that activates transcription from κB elements.

Genes differentially expressed in the PFC in alcoholics were previously pooled into 16 groups according to their functions associated with apoptosis, immune/stress response, myelination and gene transcription [14]. Genes in 14 out of 16 groups have putative NF-κB binding sites (Table S5). Among the upregulated genes in alcoholics is the death–associated protein 6 (*DAXX*). It is involved in cell survival/cell death and spatial learning processes. Interestingly, DAXX acts as an anti-apoptotic regulator [57] through its ability to repress gene transcription activated by NF-κB and other transcription factors [58]. Overall, bioinformatic data support the notion of a critical role of adaptations in the NF-κB/p50 system in the differential regulation of gene expression in the PFC of chronic alcoholics.

Tight spatio-temporal co-expression of genes functioning in the same cell-biological process is a widespread phenomenon in eukaryotes [59], [60]. Synexpression groups have been found to be coordinated by *trans*-acting factors regulating common promoter elements [59], [60]. Strong correlation between content of *RELA, NFKB1, IKKβ* and *IκBα* mRNAs suggests common regulation of transcription for these four genes. This mechanism appears to operate in the PFC but not in the MC where no strong correlations between these four mRNAs were observed.

Several animal studies suggest that chronic alcohol intake alters CNS immune and inflammatory responses through interference with NF-κB and expression of NF-κB-controlled genes [61]–[64]. Thus, chronic alcohol administration up-regulates inflammatory mediators in the animal brain and isolated astrocytes followed by activation of NF-κB and the upregulation of inducible NO synthase and cyclooxygenase-2 expression [61], [62]. Neuroinflammation may be also promoted by serum TNFα and other cytokines elevated due to systemic and hepatic inflammation induced by alcohol drinking [63]. Serum cytokines apparently enter the brain where they activate microglia. Serum and brain-produced TNFα acting through the NF-κB system may inhibit glutamate transport and, consequently, induce a hyperglutamatergic state that contributes to increased drinking and neurodegeneration [63].

Withdrawal from alcohol results in neuronal excitation [1], [65], [66] through activation of glutamate receptors and calcium influx; both stimuli activate NF-κB [15], [16], [25]–[27]. Cycles of alcohol intoxication and withdrawal, which initially activate NF-κB, when repeated over years may lead to adaptations in NF-κB that tolerate excessive stimulation. This hypothesis is supported by animal studies demonstrating that acute ethanol administration activates the NF-κB system in the brain [67], [68], which is then downregulated when ethanol is administered for the next three weeks [67]. Another adaptation to chronic alcohol consumption is the downregulation of the p50 homodimer, a dominant κB-binding factor in human cortex. This apparently results in derepression of genes regulated through κB-elements in their promoters. These types of adaptations, ensuring a low responsiveness and/or compensatory response to chronic stimulation, differ from that observed in the immune system [69], [70], which is based on the over-expression of the inhibitory p50 subunit.

Several lines of evidence demonstrate that NF-κB plays an important role in synaptic plasticity and long-term memory [15], [16], [25]–[29]. This factor is regulated by glutamate and required for LTP/LTD [28], fear conditioning [71] and spatial memory [26], [72]. NF-κB regulates, through its effects on expression of PKA, phosphorylation of CREB that is essential for transduction of synaptic signals into the cell nucleus and eventually for learning and memory [29]. These studies imply that adaptations in NF-κB in chronic alcoholics may influence neurocognitive functions through dysregulation of expression of *PKA* and other plastic genes in neurons.

In animal studies, upregulation of ΔFosB, a product of the *Fosb* gene induced by chronic administration of virtually all drugs of abuse was found to persists for several weeks and may maintain stable expression of genes associated with drug addiction [73], [74]. Dysregulation of the NF-κB system observed in the present study is associated with chronic alcoholism, and as such represents the first example of sustained transcriptional adaptation to chronic intake of substances of abuse in the human brain. The sustained adaptations in the NF-κB system apparently represent a shift to new steady state or allostatic levels in the NF-κB/p50 activities that may underlie persistent alterations in expression of their target genes. Multiple mechanisms may ensure the stability of alterations that, hypothetically, may involve a) the feedback loop between NF-κB and IκB [75]; b) feed-back and feed-forward NF-κB/p50 interactions with other transcription factors such as DAXX [58], glucocorticoid receptors [76], SP-1 [77] and CREB [29].

In conclusion, we have found that chronic alcohol consumption downregulates NF-κB and p50 homodimer in the PFC of human brain. These adaptations may be induced through several mechanisms that a) regulate transcription of the *RELA* gene, b) maintain the stoichiometry of p65 and p50 proteins, and c) control DNA-binding of p50 homodimer possibly via its phosphorylation (Figure 6). NF-κB may be targeted through the glutamate receptors, calcium influx and oxidative processes [15], [16], [25]–[28], all affected by cycles of alcohol consumption and withdrawal. Allostasis in the NF-κB system found in the present study may ensure tolerance and/or compensatory response to excessive stimulation in chronic alcoholics. Downregulation of the inhibitory p50 homodimer apparently contributes to the enhanced expression of genes regulated through κB elements in the PFC in alcoholics. Adaptations in the NF-κB system may be essential for persistent neuroplastic changes and neurodegeneration underlying dependence to alcohol and associated impairment of cognitive functions. An important role of the NF-κB system in alcoholism is emphasized by the recent animal transcriptome meta-analysis [78] and the human genetics findings. The NF-κB signaling pathway was found to be overrepresented in genes divergent between alcohol-preferring and nonpreferring mouse genotypes suggesting the participation of this system in establishing a high level of voluntary alcohol drinking [78]. The Collaborative Study on the Genetics of Alcoholism reported evidence for association of variations in the *NFKB1* (p50) gene with the risk for alcoholism (data presented at the International Narcotic Research Conference, 2005; with permission of Dr. Howard Edenberg).

## Supporting Information

Table S1 (0.09 MB XLS)

Table S2 (0.04 MB DOC)

Table S3 (0.04 MB DOC)

Table S4 (0.03 MB XLS)

Table S5 (0.03 MB XLS)

## References

[1] Fadda F, Rossetti ZL (1998). Chronic ethanol consumption: from neuroadaptation to neurodegeneration.. Prog Neurobiol.

[2] Koob GF (2003). Alcoholism: allostasis and beyond.. Alcohol Clin Exp Res.

[3] Parsons OA, Nixon SJ (1993). Neurobehavioral sequelae of alcoholism.. Neurol Clin.

[4] Schmidt KS, Gallo JL, Ferri C, Giovannetti T, Sestito N (2005). The neuropsychological profile of alcohol-related dementia suggests cortical and subcortical pathology.. Dement Geriatr Cogn Disord.

[5] Crews FT, Buckley T, Dodd PR, Ende G, Foley N (2005). Alcoholic neurobiology: changes in dependence and recovery.. Alcohol Clin Exp Res.

[6] Sullivan EV, Pfefferbaum A (2005). Neurocircuitry in alcoholism: a substrate of disruption and repair.. Psychopharmacology (Berl).

[7] Harper CG, Daly J, Kril J (1985). Brain water in chronic alcoholics: a necropsy study.. Lancet.

[8] Kril JJ, Harper CG (1989). Neuronal counts from four cortical regions of alcoholic brains.. Acta Neuropathol (Berl).

[9] Jensen GB, Pakkenberg B (1993). Do alcoholics drink their neurons away?. Lancet.

[10] Kril JJ, Halliday GM, Svoboda MD, Cartwright H (1997). The cerebral cortex is damaged in chronic alcoholics.. Neuroscience.

[11] Agartz I, Momenan R, Rawlings RR, Kerich MJ, Hommer DW (1999). Hippocampal volume in patients with alcohol dependence.. Arch Gen Psychiatry.

[12] Flatscher-Bader T, van der Brug M, Hwang JW, Gochee PA, Matsumoto I (2005). Alcohol-responsive genes in the frontal cortex and nucleus accumbens of human alcoholics.. J Neurochem.

[13] Alexander-Kaufman K, James G, Sheedy D, Harper C, Matsumoto I (2006). Differential protein expression in the prefrontal white matter of human alcoholics: a proteomics study.. Mol Psychiatry.

[14] Liu J, Lewohl JM, Harris RA, Iyer VR, Dodd PR (2006). Patterns of gene expression in the frontal cortex discriminate alcoholic from nonalcoholic individuals.. Neuropsychopharmacology.

[15] Kaltschmidt B, Widera D, Kaltschmidt C (2005). Signaling via NF-kappaB in the nervous system.. Biochim Biophys Acta.

[16] Mattson MP (2005). NF-kappaB in the survival and plasticity of neurons.. Neurochem Res.

[17] Li Q, Verma IM (2002). NF-kappaB regulation in the immune system.. Nat Rev Immunol.

[18] Guan H, Hou S, Ricciardi RP (2005). DNA binding of repressor nuclear factor-kappaB p50/p50 depends on phosphorylation of Ser337 by the protein kinase A catalytic subunit.. J Biol Chem.

[19] Li CC, Dai RM, Chen E, Longo DL (1994). Phosphorylation of NF-KB1-p50 is involved in NF-kappa B activation and stable DNA binding.. J Biol Chem.

[20] Ghosh S, Karin M (2002). Missing pieces in the NF-kappaB puzzle.. Cell.

[21] Yamamoto Y, Gaynor RB (2004). IkappaB kinases: key regulators of the NF-kappaB pathway.. Trends Biochem Sci.

[22] Moynagh PN (2005). The NF-kappaB pathway.. J Cell Sci.

[23] Kaltschmidt C, Kaltschmidt B, Baeuerle PA (1993). Brain synapses contain inducible forms of the transcription factor NF-kappa B.. Mech Dev.

[24] Bakalkin G, Yakovleva T, Terenius L (1993). NF-kappa B-like factors in the murine brain. Developmentally-regulated and tissue-specific expression.. Brain Res Mol Brain Res.

[25] Meffert MK, Baltimore D (2005). Physiological functions for brain NF-kappaB.. Trends Neurosci.

[26] Meffert MK, Chang JM, Wiltgen BJ, Fanselow MS, Baltimore D (2003). NF-kappa B functions in synaptic signaling and behavior.. Nat Neurosci.

[27] Guerrini L, Blasi F, Denis-Donini S (1995). Synaptic activation of NF-kappa B by glutamate in cerebellar granule neurons in vitro.. Proc Natl Acad Sci U S A.

[28] O'Riordan KJ, Huang IC, Pizzi M, Spano P, Boroni F (2006). Regulation of nuclear factor kappaB in the hippocampus by group I metabotropic glutamate receptors.. J Neurosci.

[29] Kaltschmidt B, Ndiaye D, Korte M, Pothion S, Arbibe L (2006). NF-kappaB regulates spatial memory formation and synaptic plasticity through protein kinase A/CREB signaling.. Mol Cell Biol.

[30] Kaltschmidt B, Uherek M, Volk B, Baeuerle PA, Kaltschmidt C (1997). Transcription factor NF-kappaB is activated in primary neurons by amyloid beta peptides and in neurons surrounding early plaques from patients with Alzheimer disease.. Proc Natl Acad Sci U S A.

[31] Mazzeo A, Aguennouz M, Messina C, Vita G (2004). Immunolocalization and activation of transcription factor nuclear factor kappa B in dysimmune neuropathies and familial amyloidotic polyneuropathy.. Arch Neurol.

[32] Huang CJ, Nazarian R, Lee J, Zhao PM, Espinosa-Jeffrey A (2002). Tumor necrosis factor modulates transcription of myelin basic protein gene through nuclear factor kappa B in a human oligodendroglioma cell line.. Int J Dev Neurosci.

[33] Nickols JC, Valentine W, Kanwal S, Carter BD (2003). Activation of the transcription factor NF-kappaB in Schwann cells is required for peripheral myelin formation.. Nat Neurosci.

[34] Association AP (1994). Diagnostic and Statistical Manual for Mental Disorders (4^th^ edn)..

[35] Vandesompele J, De Preter K, Pattyn F, Poppe B, Van Roy N (2002). Accurate normalization of real-time quantitative RT-PCR data by geometric averaging of multiple internal control genes.. Genome Biol.

[36] Johansson S, Fuchs A, Okvist A, Karimi M, Harper C (2007). Validation of endogenous controls for quantitative gene expression analysis: application on brain cortices of human chronic alcoholics.. Brain Res.

[37] Yakovleva T, Marinova Z, Kuzmin A, Seidah NG, Haroutunian V (Available online, 2006). Dysregulation of dynorphins in Alzheimer disease.. Neurobiology of aging.

[38] Baeuerle PA, Baltimore D (1988). Activation of DNA-binding activity in an apparently cytoplasmic precursor of the NF-kappa B transcription factor.. Cell.

[39] Korner M, Rattner A, Mauxion F, Sen R, Citri Y (1989). A brain-specific transcription activator.. Neuron.

[40] Bours V, Villalobos J, Burd PR, Kelly K, Siebenlist U (1990). Cloning of a mitogen-inducible gene encoding a kappa B DNA-binding protein with homology to the rel oncogene and to cell-cycle motifs.. Nature.

[41] Merino-Martinez R, Lenhard B, Flores-Morales A (Manuscript in preparation.) Prometheus: A system to integrate gene regulation and promoters analysis using grid computing.

[42] JASPAR database.. http://mordor.cgb.ki.se/cgi-bin/jaspar2005/jaspar_db.pl.

[43] Sandelin A, Wasserman WW, Lenhard B (2004). ConSite: web-based prediction of regulatory elements using cross-species comparison.. Nucleic Acids Res.

[44] Brudno M, Do CB, Cooper GM, Kim MF, Davydov E (2003). LAGAN and Multi-LAGAN: efficient tools for large-scale multiple alignment of genomic DNA.. Genome Res.

[45] Lagan aligner.. http://lagan.stanford.edu/lagan_web/index.shtml.

[46] Geiger A, Merino-Martinez R, Norstedt G, Lenhard B, Flores-Morales A (2004). Grid Computing For The Analysis Of Regulatory Elements In Co-Regulated Sets Of Genes.. Parallel Computing Letters.

[47] Bakalkin G, Yakovleva T, Terenius L (1994). Prodynorphin gene expression relates to NF-kappa B factors.. Brain Res Mol Brain Res.

[48] Bakalkin G, Yakovleva T, Hurd YL, Nussenzweig A, Li GC (1998). Autoantigen Ku in the brain. Developmentally regulated expression and subcellular localization.. Neuroreport.

[49] Bakalkin G, Yakovleva T, Melzig M, Terenius L (1997). Long-term morphine treatment increases Ku protein DNA end-binding activity.. Neuroreport.

[50] Martone R, Euskirchen G, Bertone P, Hartman S, Royce TE (2003). Distribution of NF-kappaB-binding sites across human chromosome 22.. Proc Natl Acad Sci U S A.

[51] Chen LF, Greene WC (2004). Shaping the nuclear action of NF-kappaB.. Nat Rev Mol Cell Biol.

[52] Chen-Park FE, Huang DB, Noro B, Thanos D, Ghosh G (2002). The kappa B DNA sequence from the HIV long terminal repeat functions as an allosteric regulator of HIV transcription.. J Biol Chem.

[53] Boffelli D, McAuliffe J, Ovcharenko D, Lewis KD, Ovcharenko I (2003). Phylogenetic shadowing of primate sequences to find functional regions of the human genome.. Science.

[54] Wasserman WW, Palumbo M, Thompson W, Fickett JW, Lawrence CE (2000). Human-mouse genome comparisons to locate regulatory sites.. Nat Genet.

[55] Zhong H, May MJ, Jimi E, Ghosh S (2002). The phosphorylation status of nuclear NF-kappa B determines its association with CBP/p300 or HDAC-1.. Mol Cell.

[56] Hoberg JE, Popko AE, Ramsey CS, Mayo MW (2006). IkappaB kinase alpha-mediated derepression of SMRT potentiates acetylation of RelA/p65 by p300.. Mol Cell Biol.

[57] Salomoni P, Khelifi AF (2006). Daxx: death or survival protein?. Trends Cell Biol.

[58] Michaelson JS, Leder P (2003). RNAi reveals anti-apoptotic and transcriptionally repressive activities of DAXX.. J Cell Sci.

[59] Huang R, Wallqvist A, Covell DG (2006). Comprehensive analysis of pathway or functionally related gene expression in the National Cancer Institute's anticancer screen.. Genomics.

[60] Niehrs C, Pollet N (1999). Synexpression groups in eukaryotes.. Nature.

[61] Altura BM, Gebrewold A, Zhang A, Altura BT (2002). Role of leukocytes in ethanol-induced microvascular injury in the rat brain in situ: potential role in alcohol brain pathology and stroke.. Eur J Pharmacol.

[62] Blanco AM, Valles SL, Pascual M, Guerri C (2005). Involvement of TLR4/type I IL-1 receptor signaling in the induction of inflammatory mediators and cell death induced by ethanol in cultured astrocytes.. J Immunol.

[63] Crews FT, Bechara R, Brown LA, Guidot DM, Mandrekar P (2006). Cytokines and alcohol.. Alcohol Clin Exp Res.

[64] Davis RL, Syapin PJ (2004). Ethanol increases nuclear factor-kappa B activity in human astroglial cells.. Neurosci Lett.

[65] Grant KA, Lovinger DM (1995). Cellular and behavioral neurobiology of alcohol: receptor-mediated neuronal processes.. Clin Neurosci.

[66] Grant KA, Valverius P, Hudspith M, Tabakoff B (1990). Ethanol withdrawal seizures and the NMDA receptor complex.. Eur J Pharmacol.

[67] Rulten SL, Ripley TL, Hunt CL, Stephens DN, Mayne LV (2006). Sp1 and NFkappaB pathways are regulated in brain in response to acute and chronic ethanol.. Genes Brain Behav.

[68] Ward RJ, Zhang Y, Crichton RR, Piret B, Piette J (1996). Identification of the nuclear transcription factor NFkappaB in rat after in vivo ethanol administration.. FEBS Lett.

[69] Grundstrom S, Anderson P, Scheipers P, Sundstedt A (2004). Bcl-3 and NFkappaB p50-p50 homodimers act as transcriptional repressors in tolerant CD4+ T cells.. J Biol Chem.

[70] Hajishengallis G, Genco RJ (2004). Downregulation of the DNA-binding activity of nuclear factor-kappaB p65 subunit in Porphyromonas gingivalis fimbria-induced tolerance.. Infect Immun.

[71] Yeh SH, Lin CH, Gean PW (2004). Acetylation of nuclear factor-kappaB in rat amygdala improves long-term but not short-term retention of fear memory.. Mol Pharmacol.

[72] O'Mahony A, Raber J, Montano M, Foehr E, Han V (2006). NF-kappaB/Rel regulates inhibitory and excitatory neuronal function and synaptic plasticity.. Mol Cell Biol.

[73] Hope BT, Nye HE, Kelz MB, Self DW, Iadarola MJ (1994). Induction of a long-lasting AP-1 complex composed of altered Fos-like proteins in brain by chronic cocaine and other chronic treatments.. Neuron.

[74] Ehrlich ME, Sommer J, Canas E, Unterwald EM (2002). Periadolescent mice show enhanced DeltaFosB upregulation in response to cocaine and amphetamine.. J Neurosci.

[75] Kearns JD, Basak S, Werner SL, Huang CS, Hoffmann A (2006). IkappaBepsilon provides negative feedback to control NF-kappaB oscillations, signaling dynamics, and inflammatory gene expression.. J Cell Biol.

[76] Rosenfeld MG, Lunyak VV, Glass CK (2006). Sensors and signals: a coactivator/corepressor/epigenetic code for integrating signal-dependent programs of transcriptional response.. Genes Dev.

[77] Teferedegne B, Green MR, Guo Z, Boss JM (2006). Mechanism of action of a distal NF-kappaB-dependent enhancer.. Mol Cell Biol.

[78] Mulligan MK, Ponomarev I, Hitzemann RJ, Belknap JK, Tabakoff B (2006). Toward understanding the genetics of alcohol drinking through transcriptome meta-analysis.. Proc Natl Acad Sci U S A.

