# Supplementary material for: Neuroadaptations in Human Chronic Alcoholics: Dysregulation of the NF-κB System
Source: PLoS One. 2007 Sep 26;2(9):e930. doi: 10.1371/journal.pone.0000930 (PMC1976556; doi:10.1371/journal.pone.0000930)
Supplement: Table S2 — (0.04 MB DOC) [file pone.0000930.s002.doc]

**Table S2. Levels of the NF- κB and p50 DNA-binding activity, proteins and mRNAs in the MC of alcoholics and control subjects**

|  | Control group | Alcohol group | P-value |
| --- | --- | --- | --- |
|  | Mean ± SD | Mean ± SD |  |
| mRNA levels |  |  |  |
| *RELA* | 1.63 ± 0.40 | 1.58±0.57 | 0.38 ^c^ |
| *NFKB1* | 2.13±0.64 | 2.25±0.88 | 0.96 ^a^ |
| *IKKβ* | 4.22±1.56 | 4.57±2.35 | 0.74 ^a^ |
| *IKβα* | 1.91±0.69 | 2.20±1.24 | 0.60 ^c^ |
| *NFKB2* | 3.37±1.93 | 2.69±1.26 | 0.41 ^c^ |
| *RELB* | 1.83±0.60 | 2.61±1.55 | 0.18 ^a^ |
| Protein levels |  |  |  |
| p65 | 0.65±0.20 | 0.56±0.21 | 0.23 ^a^ |
| p50 | 0.8 ±0.45 | 0.84±0.37 | 0.69 ^c^ |
| IKKβ | 0.76±0.18 | 0.65±0.17 | 0.10 ^a^ |
| DNA binding activity |  |  |  |
| NF- κB (Act) | 61.8±21.4 | 50.9±14.7 | 0.15 ^a^ |
| NF- κB (Tot) | 59.3±20.9 | 44.1±15.8 | 0.06 ^a^ |
| (p50)_2_ | 56.8±10.9 | 51.2±14.0 | 0.29 ^a^ |

NF- κB (Act), constitutively active NF- κB; NF- κB (Tot), total NF- κB activated by 0.6% DOC. Values for DNA binding activity are presented as percent of those obtained with the internal control samples loaded on each gel (control group: n=11; alcoholic group: n=13). Protein levels are presented as relative values calculated from the optical density of p65, p50 or IKKβ bands as a percent from internal control samples and normalized to Memcode staining. mRNA levels are presented as normalized relative expression levels. Protein and mRNA levels were analyzed in 15 control subjects and 15 alcoholics. Significance of differences between groups was evaluated by ^a^ Student’s *t*-test, ^b^ covariance by multiple regression analysis, and by ^c^ nonparametric Mann Whitney U-test.
