# Supplementary material for: Neuroadaptations in Human Chronic Alcoholics: Dysregulation of the NF-κB System
Source: PLoS One. 2007 Sep 26;2(9):e930. doi: 10.1371/journal.pone.0000930 (PMC1976556; doi:10.1371/journal.pone.0000930)
Supplement: Table S3 — (0.04 MB DOC) [file pone.0000930.s003.doc]

|  | Prefrontal cortex | | | | Motor cortex | | | |
| --- | --- | --- | --- | --- | --- | --- | --- | --- |
|  | Controls | | Alcoholics | | Controls | | Alcoholics | |
|  | r | P | r | P | r | P | r | P |
| **Protein**  NF-κB (Act) vs. IKKβ | 0.76 | 0.007 | 0.77 | 0.002 | 0.48 | 0.13 | 0.45 | 0.13 |
| p65 vs. p50 | 0.64 | 0.01 | 0.50 | 0.06 | 0.016 | 0.95 | 0.41 | 0.13 |
| **mRNA** |  | | | | | | | |
| *RELA* vs. *NFKB1* | 0.93 | < 0.0001 | 0.85 | < 0.0001 | 0.23 | 0.41 | 0.37 | 0.18 |
| *RELA* vs. *IKKβ* | 0.84 | < 0.001 | 0.81 | < 0.001 | -0.13 | 0.64 | 0.19 | 0.50 |
| *NFKB1* vs*. IKKβ* | 0.84 | < 0.0001 | 0.88 | < 0.0001 | 0.72 | < 0.01 | 0.79 | < 0.01 |
| *RELA* vs. *IκBα* | 0.81 | < 0.001 | 0.84 | < 0.001 | 0.49 | 0.07 | 0.09 | 0.76 |

**Table S3. Correlations (Pearsons) of constitutively active NF-κB DNA binding activity (NF-κB (Act)) with IKKβ protein levels, p65 with p50 protein levels, and between *RELA*, *NFKB1*, *IKKβ* and *IκBα* mRNA levels**
